# *Pichia pastoris* Mut^S^ strains are prone to misincorporation of *O*-methyl-l-homoserine at methionine residues when methanol is used as the sole carbon source

**DOI:** 10.1186/s12934-016-0499-2

**Published:** 2016-06-07

**Authors:** Peter Schotte, Isabelle Dewerte, Manu De Groeve, Saskia De Keyser, Veronique De Brabandere, Patrick Stanssens

**Affiliations:** Ablynx NV, Technologiepark 21, 9052 Zwijnaarde, Belgium

**Keywords:** *Pichia pastoris*, Methionine, Modifications, Nanobody, *O*-methyl-l-homoserine

## Abstract

**Background:**

Over the last few decades the methylotrophic yeast *Pichia pastoris* has become a popular host for a wide range of products such as vaccines and therapeutic proteins. Several *P. pastoris* engineered strains and mutants have been developed to improve the performance of the expression system. Yield and quality of a recombinant product are important parameters to monitor during the host selection and development process but little information is published regarding quality differences of a product produced by different *P. pastoris* strains.

**Results:**

We compared titer and quality of several Nanobodies^®^ produced in wild type and Mut^S^ strains. Titer in fed-batch fermentation was comparable between all strains for each Nanobody but a significant difference in quality was observed. Nanobodies expressed in Mut^S^ strains contained a product variant with a Δ−16 Da mass difference that was not observed in wild type strains. This variant showed substitution of methionine residues due to misincorporation of *O*-methyl-l-homoserine, also called methoxine. Methoxine is likely synthesized by the enzymatic action of *O*-acetyl homoserine sulfhydrylase and we confirmed that Nanobodies produced in the corresponding knock-out strain contained no methoxine variants. We could show the incorporation of methoxine during biosynthesis by its addition to the culture medium.

**Conclusion:**

We showed that misincorporation of methoxine occurs particularly in *P. pastoris* Mut^S^ strains. This reduction in product quality could outweigh the advantages of using Mut strains, such as lower oxygen and methanol demand, heat formation and in some cases improved expression. Methoxine incorporation in recombinant proteins is likely to occur when an excess of methanol is present during fermentation but can be avoided when the methanol feed rate protocol is carefully designed.

**Electronic supplementary material:**

The online version of this article (doi:10.1186/s12934-016-0499-2) contains supplementary material, which is available to authorized users.

## Background

The naturally occurring, heavy-chain only antibodies in *Camelidae* and their smaller derivatives called Nanobodies have attractive properties over conventional antibodies as therapeutics [1]. These structures lack the light chains of conventional antibodies and generally show better stability and solubility. Their ease in cloning and engineering allows the generation of constructs with a variety of avidity effects and bi- or multi-specificity. Nanobodies do not require the need for complex eukaryotic post-translation modifications, such as N-glycosylation, reducing the chance of unwanted heterogeneity and immunogenicity [2]. They can easily be expressed at levels of more than 1 g l^−1^ in fed-batch fermentation using micro-organisms. For therapeutic applications, immunoglobulins must be of very high product quality. This requires homogeneity in structural terms. Low yield and lack of homogeneity will impact the economics of the production process and hence, the costs for the therapeutic overall. In recent years the methylotrophic yeast *Pichia pastoris* has received wide acceptance for the production of biopharmaceuticals [3]. The success of the *P. pastoris* expression system is partly attributed to its ability to grow to high cell densities and as such increasing its volumetric productivity. In addition, *P. pastoris* uses a specific set of inducible enzymes to assimilate methanol as the sole carbon and energy source. The first step in the methanol utilization (MUT) pathway is catalyzed by two alcohol oxidases (Aox1 and Aox2) that are strongly induced when *P. pastoris* is grown on methanol [4]. The promoter of the *AOX1* gene has become a popular tool to drive the expression of recombinant proteins.

Several strains and mutants of *P. pastoris* are described and available. They can be used to manage specific issues such as proteolytic degradation or providing humanized N-linked oligosaccharide structures [5]. A specific type of strain has a deletion of the *AOX1* gene, called Mut^S^, and is commercially available from Invitrogen (KM71H), Graz University of Technology (CBS7435Mut^S^) or Biogrammatics (BG11). Mut^S^ strains still express Aox2 but grow slower than wild type strains when methanol is used as the sole carbon source. The slow growth and lower methanol consumption of Mut^S^ strains may have some advantages at large scale processes such as a lower demand for oxygen and reduced heat formation [6, 7]. In Mut^S^ strains the force of the Aox1 promoter can be directed mainly towards recombinant protein production instead of using energy for Aox1 protein production. Nevertheless, most researchers use a wild type strain, although some researchers showed that Mut^S^ strains were advantageous for production [6, 8, 9].

The production process of a biological aims to achieve the highest possible product quality, nevertheless it is likely that small quantities of unwanted variants and product related impurities are present in the end product. These include O-glycosylation, proteolytic degradation but also oxidation, carbamylation, deamidation and aggregation could occur due to specific upstream and downstream processes [10]. Strain differences or even clonal variations can also result in a difference in product quality and heterogeneity. In this study, we compared the titer and quality of several Nanobodies expressed in wild type and in Mut^S^ strains. We observed an additional modification of Nanobodies when expressed in Mut^S^ strains due to amino acid misincorporation. We characterized the nature of the modification via RPC-HPLC and ESI-Q-TOF MS. The plausible mechanism for the formation of this specific modification was further investigated and the effect of culture media and growth conditions explored.

## Results and discussion

### Mut^S^ versus wild type *P. pastoris* as a host for nanobody production

**Fig. 1:**
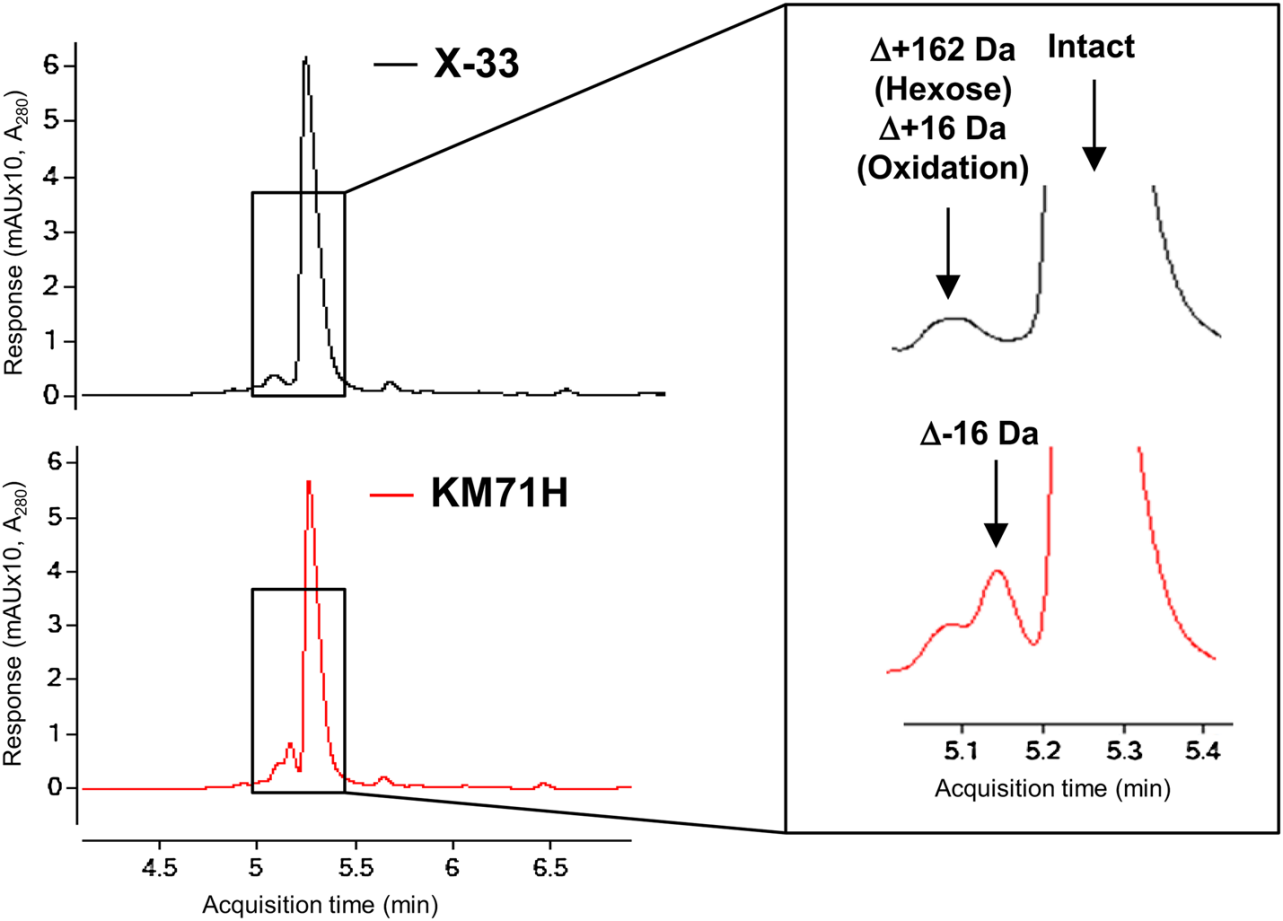
A typical RP-HPLC profile of a Nanobody and product-related variants. Purified Nanobody C produced in the wild type strain X-33 or in the Mut^S^ strain KM71H, was analysed on a reversed phase HPLC system coupled to an ESI-Q-TOF mass spectrometer. Several modifications could be identified such as oxidation (Δ+16 Da) and hexose (Δ+162 Da). An additional and unknown Δ−16 Da variant was present when Nanobody C was expressed in the Mut^S^ strain KM71H and not in the WT strain X-33. Similar observations were done with Nanobody A and B (Table 1)

To evaluate which *P. pastoris* strain is most suitable for therapeutic Nanobody development we evaluated the titer and quality of three Nanobodies produced in several wild type and Mut^S^ strains (Table 1). The strains were cultivated in 2 l fed-batch fermentations using a generic fermentation protocol. Fed-batches were performed with glycerol as carbon source followed by the methanol adaptation and induction phases. Because the Mut^S^ strains assimilate methanol more slowly than wild type, we extended the adaptation phase from 4 to 8 h, while the production phase continued for an additional 75 h. All three Nanobodies in this study are bivalent formats with a glycine–serine linker fusing the two variable domains together. Secretion of the Nanobody into the medium is controlled by the aMF secretion signal. The Nanobodies were purified using a proteinA cleanup step and expression level was determined by OD_280_ measurement. Depending on the Nanobody we observed similar expression levels in wild type and Mut^S^ strains (Table 1). The observed differences in titer between the strains for a particular Nanobody could be due to a difference in copy number of the Nanobody expression cassette. The quality was investigated by purifying the Nanobody via proteinA followed by reverse phase high performance liquid chromatography (RP-HPLC) coupled to an ESI-Q-TOF mass spectrometer. The separation is based on difference in the hydrophobicity of the Nanobody and the product-related variants, where the less hydrophobic proteins will elute earlier. Relative abundance of the Nanobody and its product-related variants is determined by comparing the integrated area of the peaks with the total integrated area. Figure 1 shows a typical RP-HPLC profile of a Nanobody and some product-related variants such as a variant with an oxidation and a variant with an O-glycosylation. Surprisingly we observed an additional product-related variant of Δ−16 Da when the Nanobodies were expressed in Mut^S^ strains but not in wild type strains. An alternative feeding procedure using a mixed carbon source of methanol and sorbitol reduced the presence of the Δ−16 Da variant significantly (Table 1). The Δ−16 Da variant was still detectable in MS but below the limit of quantification using RP-HPLC analysis.

**Table 1:** Summary table of used strains, titers in fed-batch fermentation and the % Δ−16 Da variant

Strain	Genotype	Source	Nanobody A		Nanobody B		Nanobody C	
			Titers (g l^−1^)	% Δ−16 Da variant	Titers (g l^−1^)	% Δ−16 Da variant	Titers (g l^−1^)	% Δ−16 Da variant
CBS7435 (NRRL Y-11430)	*AOX1*	ARS^a^	5.2	0	7	0		
CBS7435Mut^S^	*aox1*	[7]	4.5	4	10.5 (2.5)^a^	12 (0)^a^		
X-33	*AOX1*	Invitrogen	6.4	0	9.1	0	0.9	0
KM71H (Mut^S^)	*aox1*	Invitrogen					1.2	8

Nanobody productions were performed at 2 l scale, pH 6, 30 °C in complex medium with a methanol feed rate of 4 or 3 ml l^−1^ h^−1^ for wild type or Mut^S^ strains respectively. Except for ^a^ where the methanol feed rate was reduced to 0.5 ml l^−1^ h^−1^ in a co-feeding with sorbitol. Expression levels of Nanobodies were analyzed via a proteinA cleanup step followed by OD_280_ measurement. Relative abundance of the Δ−16 Da variant of the different Nanobodies was analysed via RP-HPLC followed by total mass measurement by ESI-Q-TOF-MS. The Δ−16 Da Nanobody variant was only observed in fed-batch fermentations with the Mut^S^ strains. A strong reduction of the Δ−16 Da was observed when the methanol feed rate was reduced to 0.5 ml l^−1^ h^−1^ and using co-feeding with sorbitol

### Identification of Δ−16 Da product-related variant produced in Mut^S^ strains

**Fig. 2:**
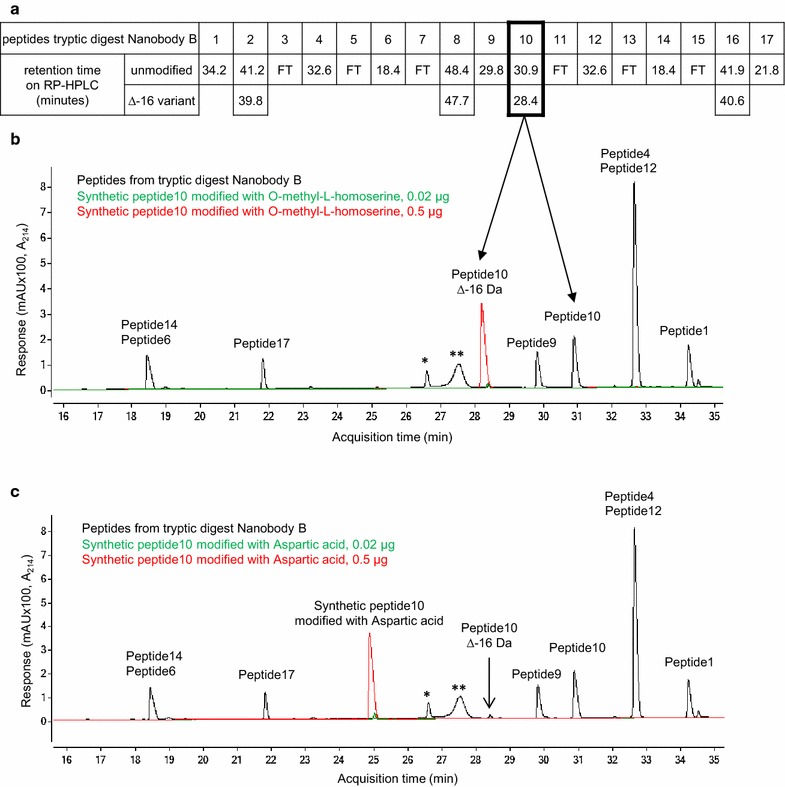
Identification of the Δ−16 Da modification as *O*-methyl-l-homoserine (methoxine) at methionine residues. **a** Tryptic digest of Nanobody B, produced and purified from the CBS7435Mut^S^ strain, generated 17 peptides. Retention times on RP-HPLC are indicated. Small peptides eluted in the flow through (FT). Four additional peptides were detected with a mass difference of −16 Da and showed a shorter retention time on RP-HPLC than their corresponding counterparts (peptides 2, 8, 10 and 16). These four peptides all contained a methionine residue. MS/MS fragmentation of the four peptides showed that the Δ−16 Da modification was located at the methionine (see Additional file 1: Figure S1); **b** and **c** Two possible amino acid substitutions resulting in a mass difference of Δ−16 Da were plausible: methionine → aspartic acid and methionine → *O*-methyl-l-homoserine. Two synthetic peptides containing these substitutions were made and their behaviour compared on RP-HPLC to the corresponding Δ−16 Da of peptide ten that was generated after trypsin digest of Nanobody B. The peptide with incorporation of *O*-methyl-l-homoserine elutes at the same retention time of the peptide that contains the Δ−16 Da modification (28.4 min) whereas the peptide containing the aspartic acid substitution showed a shorter retention time of 25.0 min (**c**). * Aspecific tryptic cleavage product of peptide 8 (see Additional file 1: Figure S1C, D); ** unknown peptide

LC–MS mass analyzer was employed to carry out MS/MS and accurate mass analysis of the specific peptides in the Nanobody that contain the Δ−16 Da modification. Nanobody B was produced in the Mut^S^ strain CBS7435Mut^S^ and contained up to 12 % of the Δ−16 Da variant. The Nanobody was purified and subjected to tryptic digest and subsequently analysed on LC–MS. A complex of 17 tryptic peptides was predicted covering the complete Nanobody sequence (Fig. 2). Nanobody B is a bivalent construct containing similar stretches in the sequence which sometimes results in the presence of twice the same peptide and consequently the same retention time on RP-HPLC. The Δ−16 Da variant was present in four peptides and, remarkably, all of them contained a methionine residue, indicating that methionine could be the possible site of the Δ−16 Da modification (Fig. 2). This was confirmed by MS/MS fragmentation of the four peptides which showed that the Δ−16 Da modification was randomly distributed at the methionine residues of the Nanobody (see Additional file 1: Figure S1). Variants with more than one Δ−16 Da modification may be present at quantities lower than the detection limit of our equipment.

Given the information that the Δ−16 Da is located at methionine residues we followed two hypotheses to identify the nature of this modification. Firstly, the misincorporation of aspartate for methionine fits a difference of Δ−16 Da. However, this is an unusual event not previously described and difficult to relate to the Mut^S^ phenotype. Secondly, Δ−16 Da is the exact mass difference between oxygen and sulphur. Changing the sulphur atom in methionine to oxygen generates *O*-methyl-l-homoserine also called methoxine. The accumulation of methoxine was previously demonstrated in several methylotrophic bacteria [11]. Therefore it is plausible that *P. pastoris* synthesizes methoxine during the induction phase when methanol is added. Moreover, Murooka et al. showed that the enzyme *O*-acetyl homoserine sulfhydrylase from *Saccharomyces cerevisiae*, which operates in the methionine biosynthesis pathway, can synthesise *O*-alkyl-l-homoserine from alcohols and *O*-acetyl-l-homoserine [12]. To analyse both hypotheses we used two synthetic peptides containing either an aspartic acid or a methoxine at the original methionine position of one of the peptides generated from the tryptic digest of Nanobody B (Fig. 2b). Due to the significant differences in hydrophobicity the two peptides will behave differently on RP-HPLC versus the methionine containing peptide. The overlay of UV 214 nm chromatograms shows that the peptide with methoxine incorporation elutes at the same retention time of the peptide that contains the Δ−16 Da modification (28.4 min) whereas the peptide containing the aspartic acid substitution is less hydrophobic resulting in a shorter retention time of 25.0 min on the column (Fig. 2c). These observations strongly suggest that the Δ−16 Da modification is the result of methoxine misincorporation at methionine residues.

### Mechanism of methoxine incorporation in *P. pastoris*

**Fig. 3:**
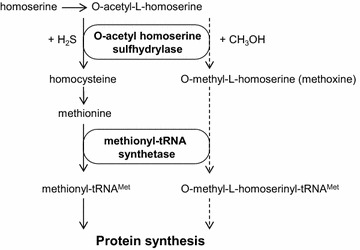
Model of methoxine incorporation in *P. pastoris*. Promiscuity of *O*-acetyl homoserine sulfhydrylase for H_2_S and methanol. *Dashed arrows* show the steps leading to the production of methoxine and subsequent incorporation into the proteome of *P. pastoris*

The methionine biosynthetic pathways of bacteria, yeast, and plants are identical up to homoserine and from homocysteine to methionine. However, from homoserine to homocysteine, the pathways differ in a number of aspects [13]. An important variation is the sulphur atom assimilation into the methionine backbone. There are two alternative routes: the first is the transsulfurylation pathway and the second the sulfhydrylation pathway. The transsulfuration pathway uses cysteine as the sulfur donor to incorporate into *O*-acetyl homoserine to form cystathionine. In the second route, the sulfur donor is sulfide, which is incorporated into *O*-acetyl homoserine by *O*-acetyl homoserine sulfhydrylase to form homocysteine. This direct sulfhydrylation pathway is found in yeast such as *S. cerevisiae* and most likely used in *P. pastoris* as well [14]. As mentioned in the above chapter the research of Murooka et al. gave us an important clue that the enzyme *O*-acetyl homoserine sulfhydrylase may be directly involved in the generation of methoxine in *P. pastoris*. A scheme detailing the role of *O*-acetyl homoserine sulfhydrylase and how incorporation of methoxine in proteins may occur is shown in Fig. 3. The assembly of methoxine into proteins reflects the imperfect selectivity of the methionyl-tRNA synthetase for methionine. This has been demonstrated previously for several analogues of methionine such as selenomethionine and telluromethionine [15], norleucine [16], trifluoromethionine [17] and ethionine [18]. To our knowledge the translational activity of methoxine has not been previously demonstrated in vivo. The fact that Mut^S^ strains are more susceptible to methoxine incorporation makes sense because the deletion of the *AOX1* gene results in lower methanol assimilation and possibly to higher intracellular methanol concentrations than in wild type strains. In the excess of methanol the intracellular concentration of methoxine might increase even further because the synthesis from its precursor *O*-acetyl homoserine only involves a single enzymatic step whereas the formation of methionine from the same precursor involves a two-step enzymatic process. In addition, induction of high Nanobody expression levels places a large demand for methionine on the cell which may result in a depletion of methionine and increased expression of the methionine biosynthetic enzymes such as *O*-acetyl homoserine sulfhydrylase, further increasing methoxine formation. It remains unclear if the formation of methoxine by the enzyme *O*-acetyl homoserine sulfhydrylase is physiologically relevant such as to remove potentially toxic excess of alcohols.

### Controlling the methoxine incorporation

**Fig. 4:**
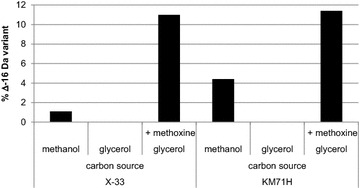
Exogenous supplied methoxine is incorporated in vivo and acts as a metabolic analogue of methionine in *P. pastoris*. Nanobody B was produced under the control of the constitutive Gap promoter in the wild type X-33 strain and in the KM71H strain (Mut^S^). Expressions were performed in shake flasks. Methoxine was added to the medium every 8 h (200 mg l^−1^) over a 48 h period. Relative abundance of the Δ−16 Da variant was done via RP-HPLC analysis followed by total mass measurement using ESI-Q-TOF-MS

To illustrate the dependency of methanol for the formation and incorporation of methoxine we analysed methoxine incorporation when glycerol or methanol was used as carbon source (Fig. 4). Therefore, the expression of Nanobody B was placed under the control of a constitutive Gap promoter. As expected, using the Mut^S^ strain KM71H as host, a significant amount of methoxine incorporation could be observed when methanol was used as carbon source. In contrast, using glycerol as carbon source we did not observe any methoxine incorporation, which illustrates the dependency of methanol in methoxine biosynthesis. Surprisingly, we observed a small amount of methoxine incorporation using the wild type strain X-33, but again, only when methanol was used as carbon source (Fig. 4). Although we never observed methoxine incorporation in Nanobodies using wild type *P. pastoris* in fed-batch fermentation, we consistently did so when expression was performed in shake flask. We believe this is related to the spiked and uncontrolled addition of methanol in shake flask whereas in fed-batch fermentation the methanol feed rate is controlled by monitoring the dissolved oxygen of the culture. This illustrates the importance of optimizing the methanol feed protocol to provide high product quality.

**Fig. 5:**
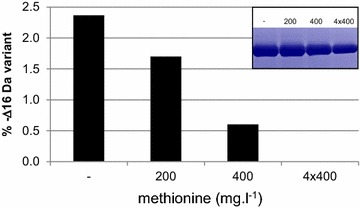
Methionine supplementation reduces the amount of the methoxine incorporation. Nanobody B was produced in the CBS7435Mut^S^ strain in shake flasks. Methionine was added at the indicated concentration when cells were switched to methanol containing medium. In one condition (4 × 400) a methionine concentration of 400 mg ml^−1^ was added four times (every 12 h after the cells were switched to methanol containing medium). Relative abundance of the Δ−16 Da variant was done via RP-HPLC analysis followed by total mass measurement by ESI-Q-TOF-MS. Expression was verified on SDS-PAGE and Coomassie staining

To show the translational activity of methoxine we added methoxine to the medium of cells that constitutively expressed Nanobody B (Fig. 5). To prevent the incorporation of endogenous produced methoxine we used glycerol as carbon source. We observed that over 10 % of Nanobody B had incorporated the exogenous supplemented methoxine. Similar levels of methoxine incorporation were observed in both wild type and Mut^S^ strains. These results support the idea that the mechanism of methoxine incorporation follows the route of mischarged methionyl-tRNA^Met^ and subsequent incorporation into proteins. Mischarging of methionyl-tRNA^Met^ and consequent misincorporation of norleucine has been described frequently during recombinant protein production in *Escherichia coli* [16, 19]. The incorporation of norleucine into peptides is the result of the inadequate substrate specificity of methionyl-tRNA synthases and evading the translational proofreading activities [20]. Norleucine can substitute for methionine at random positions in proteins which is in agreement with our observations of methoxine incorporation [21]. These misincorporations can be suppressed by addition of methionine into the medium. To evaluate if supplementation of methionine to the *P. pastoris* medium could also prevent methoxine incorporation we performed shake flask experiments with the Mut^S^ strain CBS7435Mut^S^ expressing Nanobody B (Fig. 5). The Nanobody production was initiated by switching the cells into methanol containing medium with or without methionine. The highest dose of methionine (400 mg ml^−1^) resulted in a fivefold decrease in methoxine incorporation. Supplementation of methionine every 12 h after induction prevented the methoxine incorporation completely. The level of methoxine incorporation was significantly lower in shake flask than in fed-batch fermentation (2.5 versus 12 % respectively). This could be related to the higher cell density in fermentation versus shake flask (OD_600_ = 400 versus OD_600_ = 40) resulting in an effective depletion of methionine.

To illustrate the role of the enzyme *O*-acetyl homoserine sulfhydrylase in the production of methoxine we knocked-out the enzyme in the strain CBS7435Mut^S^ expressing Nanobody B. The cells were grown in rich media containing yeast extract and peptone to balance the methionine auxotrophy. The control strain produced 0.9 ± 0.17 % methoxine variant of Nanobody B whereas the knock-out strain showed a complete absence of methoxine incorporation as analysed on RP-HPLC/MS (Table 2). This observation clearly point towards the central role of *O*-acetyl homoserine sulfhydrylase in the formation of methoxine *in P. pastoris*.

**Table 2:** Methoxine incorporation in the Mut^S^ and *O*-acetyl homoserine sulfhydrylase knock-out strains

Strain	Genotype	Source	Nanobody B	
			Titers (g l^−1^)	% Δ−16 Da variant
CBS7435Mut^S^	*aox1*	[7]	2.4 ± 0.7	0.90 ± 0.17
CBS7435Mut^S^ΔOAHS	*aox1 met25*	This study	2.1 ± 0.2	0

Nanobody B was produced in the CBS7435Mut^S^ and in the CBS7435Mut^S^ strain with an inactivation of *O*-acetyl homoserine sulfhydrylase gene (CBS7435Mut^S^ΔOAHS) in shake flasks using complex media. Titers of Nanobody B were analyzed via a proteinA cleanup step followed by OD_280_ measurement. Relative abundance of the methoxine variant was analysed via RP-HPLC followed by total mass measurement by ESI-Q-TOF-MS. The methoxine variant of Nanobody B was only produced in the Mut^S^ strain and absent when *O*-acetyl homoserine sulfhydrylase was inactivated. Experiments were performed in triplicate

## Conclusions

In this study we observed a new product related variant with a mass difference of Δ−16 Da. The modification was identified as the misincorporation of *O*-methyl-l-homoserine (also called methoxine) at methionine residues. Because several *P. pastoris* produced biopharmaceutical products have been approved for human use, one would expect to find earlier reports describing this modification. However, most researchers probably use wild type *P. pastoris* strains, which, as we demonstrated, are less likely to incorporate methoxine. Some studies have shown Mut^S^ strains to be superior over wild type strains in terms of recombinant protein production [6]. In case a Mut^S^ strain would be favourable than the wild-type strain it is important to monitor methoxine incorporation and optimize a methanol feed strategy accordingly. However, we showed that it is not impossible to have small quantities of methoxine incorporation by wild-type *P. pastoris* as well. Similar to other post-translationally modified proteins, methoxine containing proteins could elicit an immune response in humans. A proper balance between amino acid synthesis and protein synthesis would keep any detrimental effects of methoxine formation within acceptable limits. We have not investigated if the replacement of methionine with methoxine affects the activity of Nanobodies. This may depend on the position of the methionine residue or may not affect the activity at all as described for the replacement of methionine with norleucine in several enzymes [22, 23]. Methionine-rich proteins that are overproduced in *P. pastoris* using methanol as sole carbon source are probably more likely to be contaminated with methoxine residues. A purification protocol that removes all methoxine containing variants could be a difficult and costly process. Supplementation of the cultivation media with methionine could be an elegant method to produce proteins free of methoxine. The addition of methionine also represses the synthesis of the enzymes involved in methionine metabolism, including *O*-acetyl homoserine sulfhydrylase [24]. A genetic approach to prevent methoxine synthesis and incorporation could be the deletion of genes involved in the biosynthesis of methionine and methoxine such as we demonstrated with the *O*-acetyl homoserine sulfhydrylase knock-out. A straightforward way to avoid the methoxine issue is the use of methanol independent promoters such the constitutive Gap promoter, the recently developed “methanol-independent” Aox1 promoter variants [25] or glucose-limit inducible promoters [26]. Finally, it is well known that the growth speed of a Mut^S^ strain is slower than the wild type strain when grown on methanol. This has always been attributed to the slower methanol assimilation but it cannot be ruled out that this is actually to be attributed to methoxine incorporation into the proteome.

## Methods

### Strains, strain construction and vectors

*Pichia pastoris* strains used and constructed during this study are based on the wild-type strain CBS7435 (NRRL-Y11430, ATCC 76273) and described in Table 1. The strain CBS7435Mut^S^ was engineered and described by Näätsaari et al. [7]. Recombinant DNA manipulations were performed using the Top10 *E. coli* strain (Invitrogen Corp., Carlsbad, CA) according to standard protocols. All three Nanobody constructs were bivalent construct that linked two different Nanobody building blocks together with a 9GS linker. *Pichia pastoris* competent cells were prepared and transformed by electroporation as previously described [27]. Transformation of *P. pastoris* was done using 2 μg BsiWI linearized plasmid DNA harboring the Nanobodies expression cassettes of interest. Transformations were selected on YPD agar plates supplemented with 500 mg l^−1^ zeocin. *Pichia pastoris* strain CBS7435Mut^S^ was transformed with Nanobodies cloned in pPpT4_Alpha_S vector [7]. Other *P. pastoris* strains were transformed with Nanobodies cloned in the pPicZalpha or pGAPZa vector (Invitrogen). Secretion of the Nanobodies was achieved by fusion to the prepro-signal sequence of the *S. cerevisiae* alpha mating factor. The gene coding for *O*-acetyl homoserine sulfhydrylase (CAY71572.1), designated Met25, was disrupted in the strain CBS7435Mut^S^ expressing Nanobody B by gene inactivation through single homologous recombination. The knock-in vector was generated containing the last 107 base pairs of the promoter followed by the coding sequence of *O*-acetyl homoserine sulfhydrylase corresponding to the protein sequence of Met1 to Lys147. An additional base pair was inserted in the coding region of Pro2 (cct → cgct) which introduced a frameshift to prevent synthesis of a truncated *O*-acetyl homoserine sulfhydrylase. The gene fragment was synthesized using gBlocks^®^ (Integrated DNA Technologies, Inc.), amplified using the FW and RV primer set ATTATAGATCTTCAACATTGAAACCCCTCG and TCAGAAAGATCTTTATCACTTGGTCTTATCATCG and cloned via BglII into a vector carrying blasticidin resistance. The vector was linearized using the unique BamHI site in the gene fragment and transformed to the CBS7435Mut^S^ expressing Nanobody B. Clones with an inactivated *O*-acetyl homoserine sulfhydrylase gene were selected as described previously [28] and disruption of the gene was confirmed by PCR using the primer couple CCTTGCCGCATCACGTGACCCGAT which is located upstream of the promoter of the *O*-acetyl homoserine sulfhydrylase gene and GGATTGGGTGTGATGTAAGGATTC located on the backbone of the knock-out vector.

### Cultivations

Single colonies were transferred to 5 ml falcon tubes for standard cultivation as described previously [7]. The cells were cultivated for 2 days and 25 µl methanol was added to the culture in the morning and evening to maintain induction. The supernatant was then harvested and analyzed on NuPage Bis-Tris Gel (Life Technologies). The culture conditions in shake flasks for the experiment that included the *P. pastoris* strain with an inactivated *O*-acetyl homoserine sulfhydrylase were done at 30 °C in 5 ml of BGCM medium (5 g/l yeast extract, 10 g/l peptone, 10 % glycerol, 13.4 g/l YNB, 0.4 mg/l D-Biotine, 0.1 M potassium phosphate buffer pH 6). Cells were harvested by centrifugation and resuspended in 5 ml of BMCM medium which is the same as BGCM with 0.5 % (v/v) methanol instead of glycerol. The cells were cultivated for 2 days and 25 µl methanol was added to the culture in the morning and evening to maintain induction. Fed-batch cultivation were performed at 2 l scale as previously described [29]. The methanol adaptation phase for wild type strains was stepwise using a methanol feed rate of 1.5 ml l^−1^ h^−1^ for the first 2 h; 3 ml l^−1^ h^−1^ for the next 2 h followed by a feed rate at 4 ml l^−1^ h^−1^ till end of fermentation. For Mut^S^ strains the stepwise methanol adaptation phase was from 0 to 2 h at 1 ml l^−1^ h^−1^ followed by a feed rate at 3 ml l^−1^ h^−1^ till end of fermentation. Mixed feeding was performed during the induction phase using 0.5 ml l^−1^ h^−1^ methanol with 4.75 g l^−1^ h^−1^ of a 60 % (w/v) sorbitol solution.

### Chemicals

Enzymes were purchased from New England Biolabs and trypsin from Promega. Difco™ yeast nitrogen base without amino acids (YNB), HypA peptone was obtained from BioSpringer and yeast extract from Oxoid. Glucose was obtained from Merck chemicals, glycerol from VWR chemicals, sorbitol and D-Biotin from Sigma Aldrich. Select agar and Zeocin™ were obtained from Invitrogen. Methoxine (*O*-methyl-l-homoserine) was supplied by IRIS Biotech GmbH and methionine ordered at Sigma-Aldrich. Synthetic peptides were ordered at Bachem.

### Analysis

ProteinA cleanup was performed using MabSelect Xtra resin (GE Healthcare). Briefly clarified broth sample was centrifuged to remove any remaining particulate matter. The sample was applied on the column according to the manufacturer’s instructions. The column was washed with six column volumes of D-PBS buffer before elution with 1 ml of 0.1 % TFA. Based on the protein concentration obtained by UV absorbance (Jasco V-650), approximately 4 μg of each elution sample was analyzed on RP-HPLC/MS. The presence of Nanobody related variants was analyzed using a Reversed Phase HPLC system (Agilent) coupled to an ESI-Q-TOF mass spectrometer. Relative abundance of variants was performed on baseline to valley integration. Trypsin digest of Nanobody B was performed with 1:25 of Trypsin: Nanobody (w:w) and 0.1 % Rapigest in 100 mM Tris-HCl pH 7.5 and incubated at 37 °C. Of this digest 7.5 μg was applied on an Acquity UPLC BEH column from Waters combined with Agilent Q-TOF to perform the MS/MS analysis.

## Additional file

10.1186/s12934-016-0499-2 MS/MS fragmentation of four peptides indicates that the Δ−16 Da modification was located at methionine residues. Typical peptide fragmentation generates b or y ions of different mass to charge ratios. Correspondence of the experimentally determined masses to the molecular masses of the amino acid residues can be used to derive the sequence of the parent ion. Fragmentation by collisional-induced dissociation of the peptides 2, 10 and 16 showed via the b and/or y ions that the modification of Δ−16 Da is indeed located at the methionine residue in all peptides. The fragmentation of peptide 8 was unsuccessful. However, an aspecific tryptic cleavage product of peptide 8 could be used for the fragmentation and the b and y ions showed again that the modification of Δ−16 Da is located at the methionine residue. A, C, E, G Show the theoretical spectral fragments of each peptide; B, D, F, H, show the observed spectral fragmentation of each peptide.

## Abbreviations

Aox: alcohol oxidase
Gap: glyceraldehyde 3-phosphate
Mut^S^: methanol utilization slow (knock-out of Aox1)
Oahs: *O*-acetyl homoserine sulfhydrylase
RP-HPLC: reverse phase high performance liquid chromatography
ESI-Q-TOF MS: electrospray ionization-quadrupole-time of flight mass spectrometry
UPLC: ultra-high pressure liquid chromatography
