# Supplementary material for: Pichia pastoris MutS strains are prone to misincorporation of O-methyl-l-homoserine at methionine residues when methanol is used as the sole carbon source
Source: Microb Cell Fact. 2016 Jun 7;15:98. doi: 10.1186/s12934-016-0499-2 (PMC4897801; doi:10.1186/s12934-016-0499-2)
Supplement: Supplementary file 1 — 10.1186/s12934-016-0499-2 MS/MS fragmentation of four peptides indicates that the Δ−16 Da modification was located at methionine residues. Typical peptide fragmentation generates b or y ions of different mass to charge ratios. Correspondence of the experimentally determined masses to the molecular masses of the amino acid residues can be used to derive the sequence of the parent ion. Fragmentation by collisional-induced dissociation of the peptides 2, 10 and 16 showed via the b and/or y ions that the modification of Δ−16 Da is indeed located at the methionine residue in all peptides. The fragmentation of peptide 8 was unsuccessful. However, an aspecific tryptic cleavage product of peptide 8 could be used for the fragmentation and the b and y ions showed again that the modification of Δ−16 Da is located at the methionine residue. A, C, E, G Show the theoretical spectral fragments of each peptide; B, D, F, H, show the observed spectral fragmentation of each peptide. [file 12934_2016_499_MOESM1_ESM.pptx]

## Slide 1
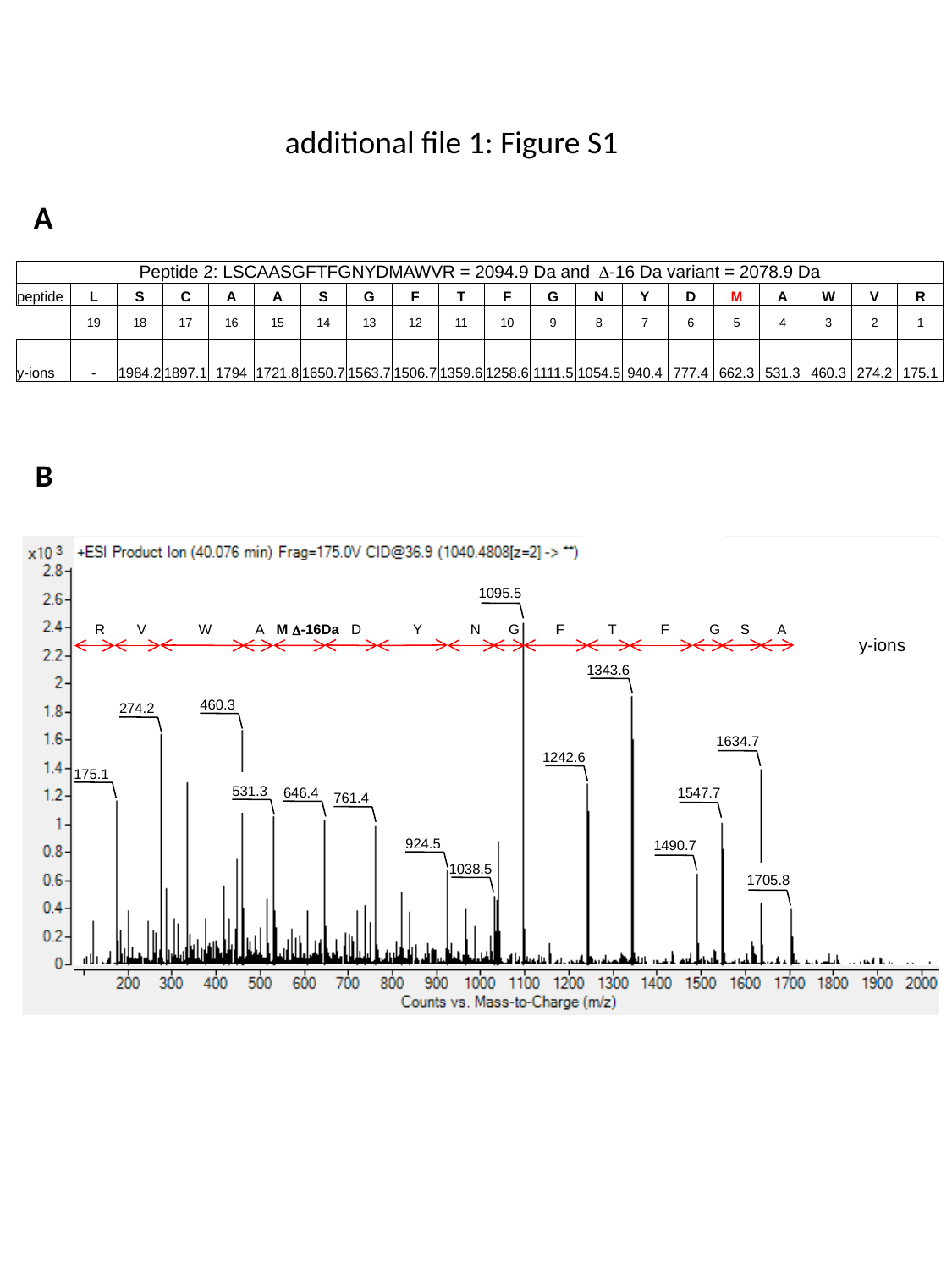

additional file 1: Figure S1
A
| Peptide 2: LSCAASGFTFGNYDMAWVR = 2094.9 Da and D-16 Da variant = 2078.9 Da | | | | | | | | | | | | | | | | | | | |
| --- | --- | --- | --- | --- | --- | --- | --- | --- | --- | --- | --- | --- | --- | --- | --- | --- | --- | --- | --- |
| peptide | L | S | C | A | A | S | G | F | T | F | G | N | Y | D | M | A | W | V | R |
| | 19 | 18 | 17 | 16 | 15 | 14 | 13 | 12 | 11 | 10 | 9 | 8 | 7 | 6 | 5 | 4 | 3 | 2 | 1 |
| y-ions | - | 1984.2 | 1897.1 | 1794 | 1721.8 | 1650.7 | 1563.7 | 1506.7 | 1359.6 | 1258.6 | 1111.5 | 1054.5 | 940.4 | 777.4 | 662.3 | 531.3 | 460.3 | 274.2 | 175.1 |
B
1095.5
 R V W A M D-16Da D Y N G F T F G S A
y-ions
1343.6
460.3
274.2
1634.7
1242.6
175.1
761.3886
531.3
1547.7
646.4
761.4
924.5
1490.7
1038.5
1705.8

## Slide 2
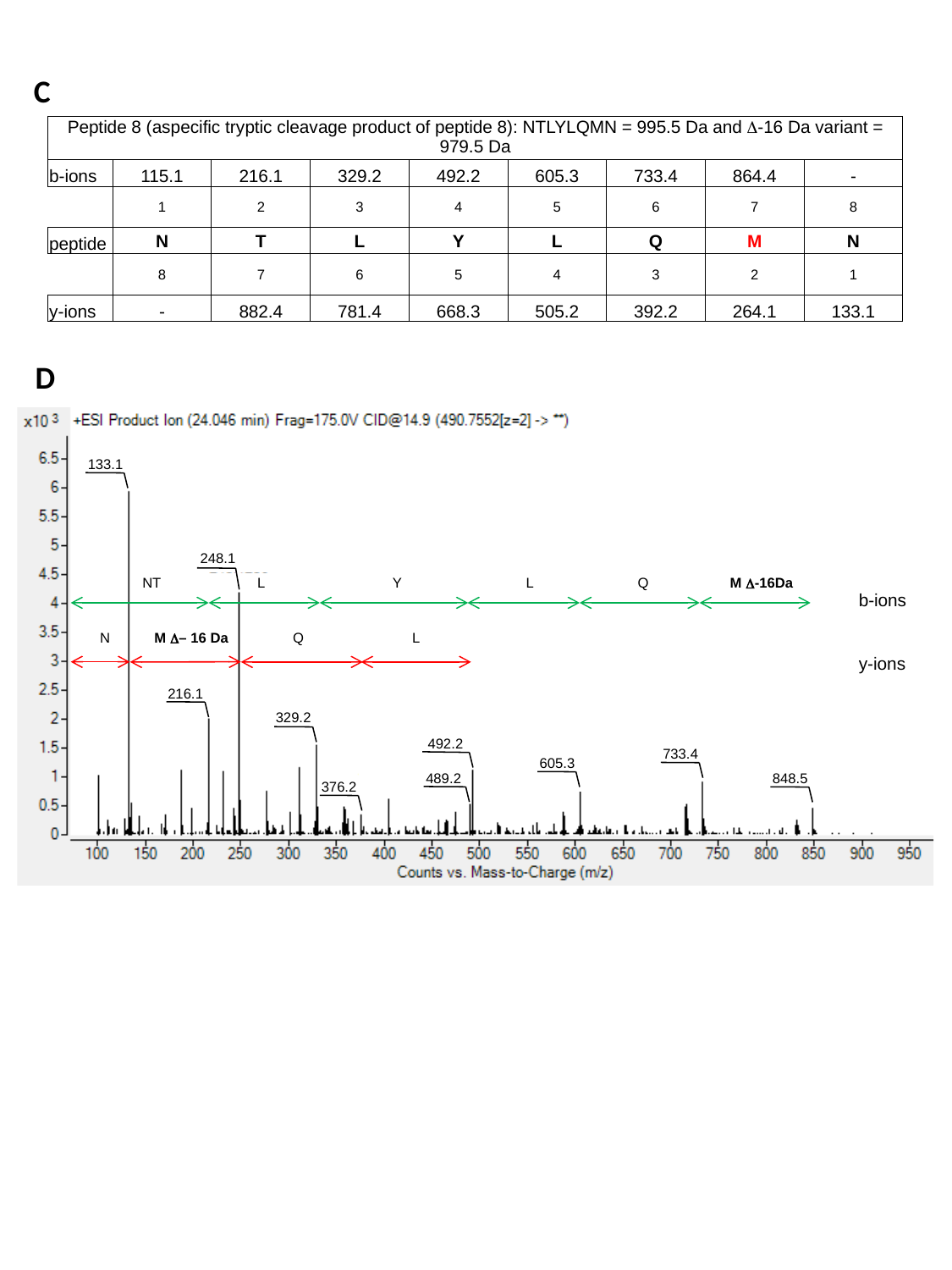

C
| Peptide 8 (aspecific tryptic cleavage product of peptide 8): NTLYLQMN = 995.5 Da and D-16 Da variant = 979.5 Da | | | | | | | | |
| --- | --- | --- | --- | --- | --- | --- | --- | --- |
| b-ions | 115.1 | 216.1 | 329.2 | 492.2 | 605.3 | 733.4 | 864.4 | - |
| | 1 | 2 | 3 | 4 | 5 | 6 | 7 | 8 |
| peptide | N | T | L | Y | L | Q | M | N |
| | 8 | 7 | 6 | 5 | 4 | 3 | 2 | 1 |
| y-ions | - | 882.4 | 781.4 | 668.3 | 505.2 | 392.2 | 264.1 | 133.1 |
D
133.1
248.1
NT L Y L Q M D-16Da
b-ions
y-ions
 N M D– 16 Da Q L
216.1
329.2
492.2
733.4
605.3
848.5
489.2
376.2

## Slide 3
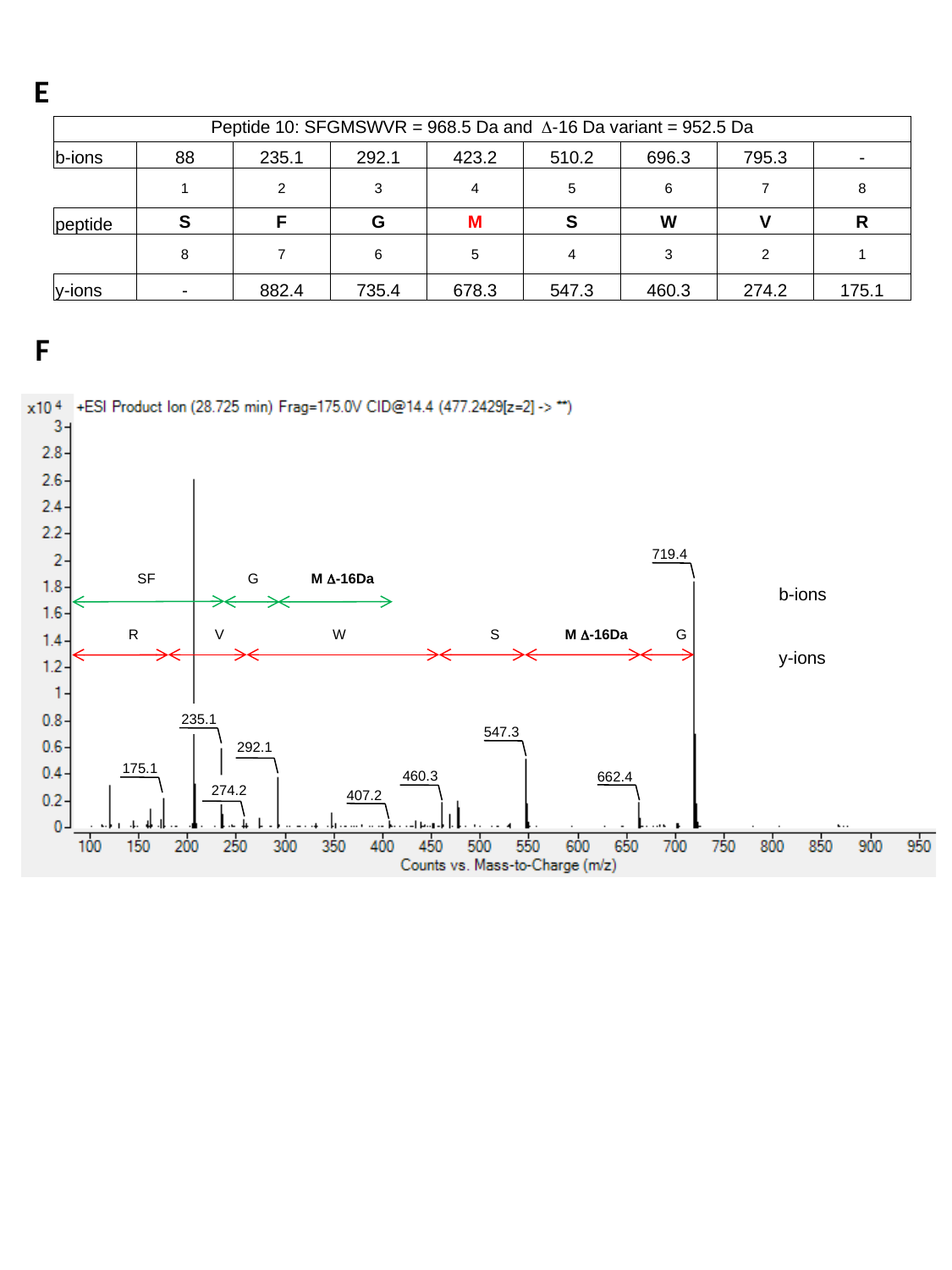

E
| Peptide 10: SFGMSWVR = 968.5 Da and D-16 Da variant = 952.5 Da | | | | | | | | |
| --- | --- | --- | --- | --- | --- | --- | --- | --- |
| b-ions | 88 | 235.1 | 292.1 | 423.2 | 510.2 | 696.3 | 795.3 | - |
| | 1 | 2 | 3 | 4 | 5 | 6 | 7 | 8 |
| peptide | S | F | G | M | S | W | V | R |
| | 8 | 7 | 6 | 5 | 4 | 3 | 2 | 1 |
| y-ions | - | 882.4 | 735.4 | 678.3 | 547.3 | 460.3 | 274.2 | 175.1 |
F
719.4
 SF G M D-16Da
b-ions
y-ions
 R V W S M D-16Da G
235.1
547.3
292.1
175.1
460.3
662.4
274.2
407.2

## Slide 4
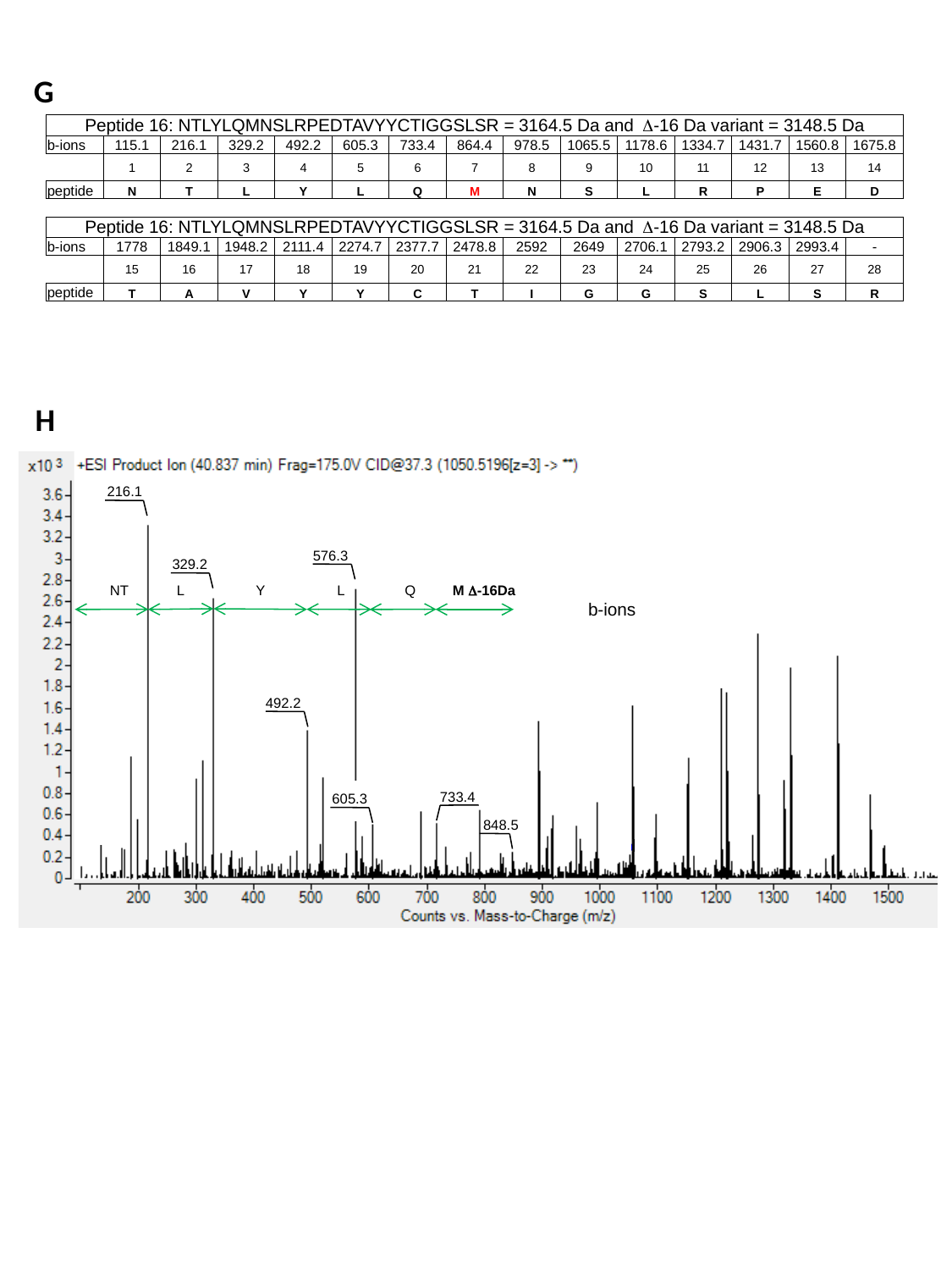

G
| Peptide 16: NTLYLQMNSLRPEDTAVYYCTIGGSLSR = 3164.5 Da and D-16 Da variant = 3148.5 Da | | | | | | | | | | | | | | |
| --- | --- | --- | --- | --- | --- | --- | --- | --- | --- | --- | --- | --- | --- | --- |
| b-ions | 115.1 | 216.1 | 329.2 | 492.2 | 605.3 | 733.4 | 864.4 | 978.5 | 1065.5 | 1178.6 | 1334.7 | 1431.7 | 1560.8 | 1675.8 |
| | 1 | 2 | 3 | 4 | 5 | 6 | 7 | 8 | 9 | 10 | 11 | 12 | 13 | 14 |
| peptide | N | T | L | Y | L | Q | M | N | S | L | R | P | E | D |
| | | | | | | | | | | | | | | |
| Peptide 16: NTLYLQMNSLRPEDTAVYYCTIGGSLSR = 3164.5 Da and D-16 Da variant = 3148.5 Da | | | | | | | | | | | | | | |
| b-ions | 1778 | 1849.1 | 1948.2 | 2111.4 | 2274.7 | 2377.7 | 2478.8 | 2592 | 2649 | 2706.1 | 2793.2 | 2906.3 | 2993.4 | - |
| | 15 | 16 | 17 | 18 | 19 | 20 | 21 | 22 | 23 | 24 | 25 | 26 | 27 | 28 |
| peptide | T | A | V | Y | Y | C | T | I | G | G | S | L | S | R |
H
216.1
576.3
329.2
 NT L Y L Q M D-16Da
b-ions
492.2
733.4
605.3
848.5
